# Identification of ERp29 as a biomarker for predicting nasopharyngeal carcinoma response to radiotherapy

**DOI:** 10.3892/or.2011.1586

**Published:** 2011-12-08

**Authors:** PING WU, HUA ZHANG, LIN QI, QINGPING TANG, YAOYUN TANG, ZHIHAI XIE, YUNXIA LV, SUPING ZHAO, WEIHONG JIANG

**Affiliations:** 1 Department of Otolaryngology, Xiangya Hospital, Central South University, Changsha 410008; 2 Department of Rehabilitation, The Second Hunan Provincial People's Hospital, Changsha 41007, Hunan, P.R. China

**Keywords:** ERp29, proteomics, radioresistance, human nasopharyngeal carcinoma

## Abstract

Radioresistance continues to be a major problem in the treatment of nasopharyngeal carcinoma (NPC). This study aimed to identify novel proteins associated with NPC radioresistance. We used a mass spectrometry driven-proteomic strategy to identify novel proteins associated with NPC radioresistance, and differential proteins were subsequently processed by bioinformatic analysis. As a result, twelve proteins were identified with aberrant expression in radioresistant (RR) NPC tissues compare to radiosensitive (RS) NPC tissues. Among these proteins, ERp29, Mn-SOD, HSP27 and GST ω1 were found to be significantly up-regulated in RR NPC tissues, and ERp29 was selected for further validation. Immunohistochemistry analysis confirmed that ERp29 was overexpressed in RR NPC tissues compared with RS NPC tissues. To prove the role of ERp29 in the induction of NPC radioresistance, ERp29 was down-regulated in the ERp29 enriched NPC cells CNE-1 and 6-10B by specific shRNA. Radiosensitivity was measured using cell proliferation assay and clonogenic survival assay, and cell apoptosis was measured using flow cytometric analysis. We found that ERp29 knockdown attenuated CNE-1 and 6-10B cell radioresistance and enhanced cell apoptosis. These results suggest that ERp29 associates with radioresistance in NPC, and ERp29 could be a potential biomarker for predicting NPC response to radiotherapy.

## Introduction

NPC is one of the most common malignant tumors in southern China and Southeast Asia with incidence rates of 20 to 50 per 100,000 (1). The most effective treatment for NPC is radiotherapy, which achieves an overall 5-year survival rate of 65% (2). Nevertheless, radioresistance remains a serious barrier to successful treatment in many cases. Radioresistance easily induces in some NPC patients local recurrence and distant metastases and the majority of these patients suffer recurrence and metastasis within 1.5 year after treatment (3,4). To obtain optimal effect of radiotherapy on NPC patients, it is urgent to identify subgroup of radioresistant NPC patients and to reveal the molecular mechanism of NPC radioresistance.

Many studies have been directed toward understanding the mechanism of radiotherapy response and resistance, with the ultimate goal of identifying molecular markers that allow the prediction of radiotherapy response. Several proteins, Epstein-Barr virus gene BHRF1 (5), antioxidant enzyme manganese superoxide dismutase (MnSOD_2_) (6), 14-3-3σ (7), and raf kinase inhibitory protein (RKIP) (8) have been proved to be associated with radioresistance in NPC. However, almost all of these proteins were originally found from RR NPC cells not from RR NPC tissues with scarcity of clinical specificity. High-throughput technology is needed which offer the potential ability to find specific RR proteins in NPC. Proteomics, which aims at identifying differential proteins associated with differential disease traits, has been proved to be an effective approach in protein research (9). To identify the proteins associated with the radioresistance of NPC, we performed comparative proteomics on RR and RS NPC tissues in order to find differential proteins associated with radioresistance.

ERp29 is an endoplasmic reticulum(ER) protein and has emerged in a variety of physiological and pathological conditions, such as normal production of dental enamel, antibodies, and milk, and disorders of the thyroid, spinal cord, and aging eye (10–13). Many studies indicated that ERp29 was expressed in a variety of human cancer and it was found to be highly expressed in primary tumors and cell lines (14–16). It was also reported that ERp29 was up-regulated in mouse intestinal epithelia cells when exposure to radiation (17), indicating ERp29 associated with resistance to radiation stress and may play a potential protective role against stress. Nevertheless, whether ERp29 is involved in radioresistance in human cancer has not yet been elucidated.

In this study, based on the results of the comparative proteomic analysis of RR and RS NPC tissues, we identified twelve differential proteins and verified ERp29 overexpression in the RR NPC tissues. Then we attempted to elucidate that ERp29 knockdown attenuated CNE-1 and 6-10B cell radioresistance and enhanced cell apoptosis.

## Materials and methods

### Patients and tissues

We selected 88 NPC patients who were treated by curative-intent radiotherapy (a total dose of 70 Gy) using a modified linear accelerator in the Xiangya Hospital of Central South University, China, from January 2003 to June 2007. NPC patients recruited in this study included 42 RR and 46 RS patients. RR NPC patients were defined as those with persistent disease (incomplete regression of tumor) at 6 weeks or more, or those with recurrent disease at the nasopharynx and/or neck nodes at 2 months or more after completion of radiotherapy (18). RS NPC patients were defined as those without local residual lesions at 6 weeks or no recurrence at 2 months after completion of radiotherapy (18). NPC tissue biopsies from these 88 patients were obtained at the time of diagnosis before any therapy with an informed consent. Ten pairs of RR and RS NPC tissues by random sampling were frozen in liquid nitrogen for proteomic analysis, and other tissues were used for immunohistochemical staining. All cases in this study were histopathologically diagnosed as poorly differentiated squamous cell carcinomas by examination of the frozen sections and paraffin imbedded sections based on the 1978 WHO classification (19). The clinicopathological parameters of 88 archival NPC tissue specimens used in the present study are shown in Table I. This study was approved by the ethics committee of Xiangya School of Medicine, Central South University, China.

### Proteomic analysis

The detailed approach of two-dimensional gel electrophoresis (2-DE), image analysis and mass spectrometer (MS) analysis was described by Cheng *et al* (20). Briefly, 10 pairs of radioresistant and radiosensitive NPC tissues were dissolved in lysis buffer (7 mol/l urea, 2 mol/l thiourea, 100 mmol/l DTT, 4% CHAPS, 0.5 mmol/l EDTA, 40 mmol/l Tris, 2% NP40, 1% Triton X-100, 5 mmol/l PMSF, and 2% phamarlyte) at 4˚C for 1 h. Then the supernatant was transferred after centrifugation at 12,000 rpm for 30 min at 4˚C. After detection of protein concentration, total proteins were separated by 2D Quantification kit (Amersham Biosciences) from twenty sets with each set containing a radioresistant or a radiosensitive NPC tissue. After Blue Silver staining, the stained 2-DE gels of each set were scanned by MagicScan software on an Image scanner (Amersham Biosciences), and analyzed using a PDQuest system (Bio-Rad Laboratories, Hercules, CA). Proteins were classified as being differentially expressed between the two types of tissues when spot intensity showed a 2-fold variation in radioresistant NPC tissue compared to radiosensitive NPC tissue. All the differential protein spots were excised from stained gels. After trypsin digestion, the mixture was analyzed by a Voyager System DE-STR 4307 MALDI-TOF mass spectrometer (MS) (ABI, Foster City, CA, USA) to get a peptide mass fingerprint (PMF). Mascot Distiller was used to obtain the monoisotopic peak list from the raw mass spectrometry files. Peptide matching and protein searches against the Swiss-Prot database were performed using the Mascot search engine (http://www.matrixscience.com/) with a mass tolerance of ±50 ppm.

### Immunohistochemistry staining

Immunohistochemistry was performed using the following protocol. Forty-two radioresistant and forty-six radiosensitive NPC tissues sections were deparaffinized in xylene. Sections were rehydrated in alcohol, and pretreated with citrate buffer (10 mmol/l, pH 6.0) for 20 min at 100˚C in a microwave oven. Endogenous peroxidase activity was blocked with 3% hydrogen peroxide for 15 min at room temperature, then nonspecific binding sites were blocked by 10% normal goat serum for 30 min at 37˚C. The sections were incubated with antibody (rabbit polyclonal anti-ERP29 1:200 dilution, Abcam) overnight at 4˚C. After washing with PBS, sections were incubated with 1:1000 dilution of biotinylated goat anti-rabbit IgG (Zhongshan Chemical) for 20 min at 37˚C. Finally, tissue sections were incubated with 3′,3′-diaminobenzidine (Maixin, Fuzhou) until a brown color emerged and washed with distilled water, then counterstained with Harris modified hematoxylin (Zhongshan Chemical). Primary antibodies were omitted for negative controls.

### Counting and statistical methods

Sections were blindly evaluated by two pathologists by light microscopy. A semi-quantitative scoring criterion for immunohistochemistry was used, in which both the intensity and the percentage of positive cells were evaluated according to the methods by Hara and Okayasu (21). More than 10 microscopic fields were chosen randomly with ×400 magnification, and >1000 cells were counted for each section. The intensity of staining was graded on the following scale: 0, no stain; 1, mild staining; 2, moderate staining; 3, intense staining. The number of positive cells was visually evaluated as follows: 0, ≤10% tissue stained positive; 1, 10 to 30% stained positive; 2, 30 to 60% stained positive; 3, >60% stained positive. The minimum score summed (extension + intensity) was therefore 0 and the maximum was 6. A combined staining score (extension + intensity) ≤2 was considered to be weak staining; a score 3 or 4 moderate; and 5 or 6 intense staining.

### Cell culture

NPC cell lines CNE-1, CNE-2, 5-8F, 6-10B used in this study were obtained from cancer institute, Central South University. NPC cells were cultured in RMPI-1640 medium (Gibco, NY, USA) supplemented with 10% of fetal bovine serum FBS (Gibco) at 37˚C in an incubator at a humidified atmosphere with 5% CO_2_ in air.

### Western blotting

Briefly, 40 μg of lysates were separated by 10% SDS-PAGE and transferred to a polyvinylidene difluoride membrane. Non-specific binding sites were blocked by 5% normal goat serum at room temperature for 1 h. The membrane was incubated with primary antibody: anti-ERP29 (Abcam, 1:2000 dilution) overnight at 4˚C. Then horseradish peroxidase conjugated secondary antibody (Beyotime, Beijing, China, 1:2000 dilution) for 1 h. The immune complexes were visualized by enhanced electrochemiluminescence (ECL) detection. The ECL test kit-based detection was performed with Chemiluminescence Reagent (Formantas biology) according to the manufacturer's instructions. β-actin was used for loading control. The results of Western blot analysis represented the average of three individual experiments.

### Clonogenic survival assay

Cells were plated in triplicate at cell population of 10^2^, 2×10^2^, 4×10^2^, 10^3^, 10^4^, 10^5^ per dish, and then were exposed to a range of radiation doses (0–8 Gy). After irradiation, the cells were cultured for no less than 12 days and the number of surviving colonies (defined as a colony with >50 cells) was counted and the data normalized to the appropriate sham-irradiated control group. Survival parameters D_0_ and N were fitted according to the linear quadratic equation [S=1−(1−e−D/Do)N] using SigmaPlot 9.0 software (Systat Software Inc., USA). Three independent experiments were done.

### Stable transfection

The ERp29-targeted shRNA lentiviral particles (sc-60599-V) and no-targeted shRNA lentiviral particles (sc-108080) as control, purchased from Santa Cruz Biotechnology (USA), were transfected into NPC cells according to the manufacturer's instructions. After 14 days of selection in RIPM-1640 containing 10% FBS and 10 μg/ml puromycin (Santa Cruz Biotechnology), individual puromycin-resistant colonies were isolated and expanded. The expression of ERp29 was determined by Western blot analysis as above described.

### Cell viability assay

2-(2-methoxy-4-nitrophenyl)-3-(4-nitrophenyl)-5-(2,4-disulfophenyl)-2H-tetrazolium, monosodium salt (CCK-8, Beyotime) assay was used to detect cell viability in response to irradiation. Briefly, cells were seeded in 96-well culture plates at 3×10^3^ for CNE-1 cells or 2×10^3^ for 6-10B cells per well. After incubation for 8 h, the cells were exposed to 8 Gy X-ray irradiation. Cell viability was determined by CCK-8 at various time intervals according to the manufacturer's instructions. Optical densities were determined on a microtiter plate reader (Peskin and Winterbourn 2000) at 450 nm. Three independent experiments were done in triplicate.

### Flow cytometry analysis of cell apoptosis

Cells were seeded in 6-well culture plates at 1×10^5^ for CNE-1 cells or 5×10^4^ for 6-10B cells per well. Then the cells were harvested at 72 h after irradiation with 8 Gy X-ray. According to the manufacturer's instructions of Annexin V-FITC apoptosis detection kit (Beyotime), cells were stained using Annexin-V-FITC for 10 min at room temperature and then were stained using propidium iodure (PI) for 10 min in dark. The cells were analyzed immediately on a FSCAN flow cytometer (BD Biosciences, USA). All samples were assayed in triplicate.

### Statistics

All statistical analyses were carried out using SPSS for Windows version 13.0 (SPSS). χ^2^ test was applied to analyze the relationship between ERp29 expression and clinicopathologic features. Student's t-test and One-way analysis of variance (ANOVA) were used to analyze the cell experimental data. Data are presented as the mean ± standard deviation (SD). Differences were considered statistically significant for P<0.05.

## Results

### Screening for radioresistance-associated proteins by proteomic analysis

**Figure 1:**
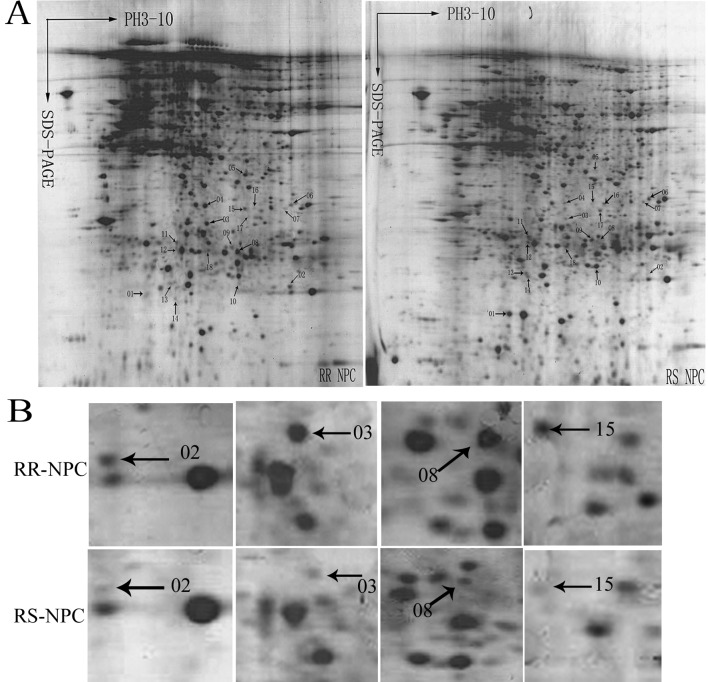
Comparative proteomic analysis of RR and RS NPC tissues. (A) Representative 2-DE maps of RR- and RS-NPC tissues, eighteen differential protein spots are marked with arrows. (B) Zoomed regions of gel images from RR and RS NPC tissues. Images of the region of 2-D gels show the upregulation of protein spot 02 (Mn-SOD), spot 03 (ERp29), spot 08 (Hsp27) and spot 15 (GST ω1) in RR NPC tissue compared with RS NPC tissue.

Comparative proteomic study of RR and RS NPC tissues was performed to identify the proteins associated with radioresistance. Ten pairs of 2-DE maps from RR NPC tissues and control RS NPC tissues were constructed with PDQuest image software. A total of 18 differential protein spots (≥2-fold) in the two types of tissues were detected, and subjected to the analysis of MAL-DI-TOF-MS. Two representative 2-DE maps from RR and RS NPC tissues are shown in Fig. 1A, and 18 differential protein spots are marked with arrows. Images of the region of the 2-DE showing spot 02 (Mn-SOD, up-regulated 3.1-fold), spot 03 (ERp29, up-regulated 4.7-fold) and spot 08 (HSP27, up-regulated 3.9-fold) in the RR NPC tissues compare to the RS NPC tissues are shown in Fig. 1B. The 18 differential protein spots were analyzed by MAL-DI-TOF-MS and 12 proteins were identified. Among these proteins, Mn-SOD, ERp29, HSP27 and GST ω1 were up-regulated in the RR NPC tissues; TSP-1, PKM2, human electron transfer flavoprotein, MRP-14, DJ-1, GMFG, prohibitin and cytochrome c oxidase were down-regulated in the RR NPC tissues. The annotation of all the identified proteins is summarized in Table II.

### Expression of ERp29 in clinical specimens and NPC cell lines

**Figure 2:**
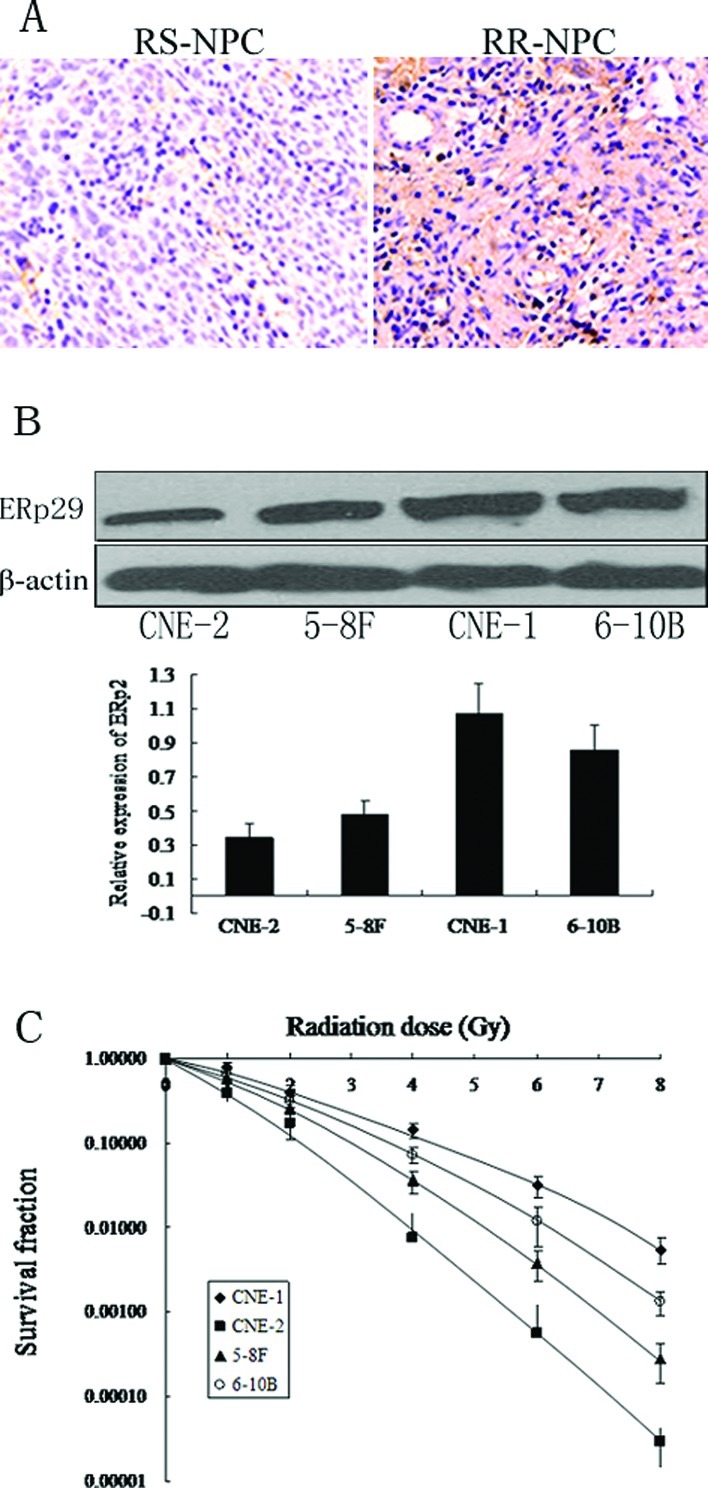
(A) Expression of ERp29 in RR- and RS-NPC tissues. A representative result of immunohistochemistry shows ERp29 evident in the cytoplasm of tumor cells and overexpressed in the RR-NPC tissues compared with the RS-NPC tissues. Original magnification, ×200. (B) A representative result of Western blot analysis shows the expression of ERp29 in NPC cell lines CNE-2, 5-8F, CNE-1 and 6-10B. β-actin was used as an internal control for loading. (C) Clonogenic survival curves of NPC cell lines CNE-2, 5-8F, CNE-1 and 6-10B, the steeper curves indicate stronger radioresistance of cells. Points, mean; bars, ±SD.

To validate the relevance of ERp29 to NPC radioresistance, we detected ERp29 expression in 42 radioresistant and 46 radiosensitive formalin-fixed paraffin-embedded biopsy samples by immunohistochemistry. ERp29 staining, when present, was evident in the cytoplasm of tumor cells (Fig. 2A). ERp29 antigen showed intense staining in the majority of radioresistant NPC tissues (57.1%) and weak staining in the majority of radiosensitive NPC tissues (67.4%). ERp29 overexpression was significantly associated with radioresistant tumors (P<0.001, Table III). To further detect the association of ERp29 expression with NPC radioresistance, we detected the ERp29 expression in NPC cell lines CNE-1, CNE-2, 5-8F and 6-10B with different radioresistant potentials. As shown in Fig. 2B, the expression of ERp29 descending order is CNE-1, 6-10B, 5-8F and CNE-2. Meanwhile, the radioresistance of NPC cell lines in descending order is also CNE-1, 6-10B, 5-8F and CNE-2 (Fig. 2C). This indicates that ERp29 expression positively correlated with radioresistance of NPC cell lines.

### ERp29 knockdown attenuated CNE-1 and 6-10B cells radioresistance

**Figure 3:**
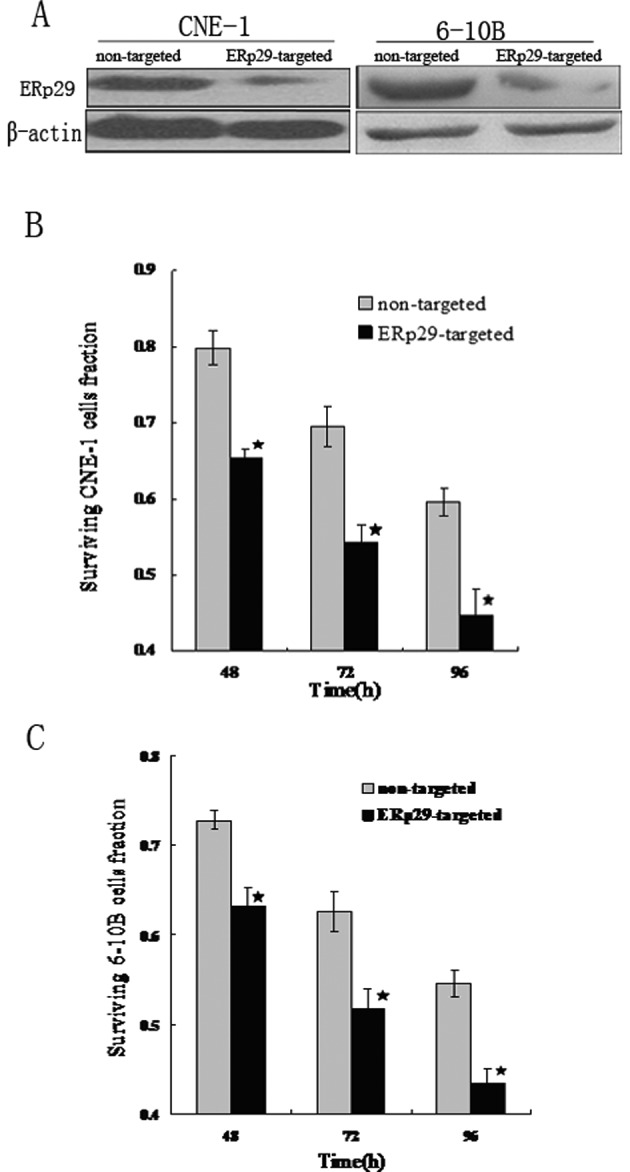
(A) A representative result of Western blot analysis shows the downregulation of ERp29 expression in ERp29-targeted shRNA transfected CNE-1 and 6-10B cells compared with non-targeted shRNA transfected cells. (B and C) Metabolic activity of non-targeted or ERp29-targeted shRNA transfected CNE-1 and 6-10B cells were determined by CCK-8 assay without (0 Gy) and 48, 72, and 96 h after irradiation with 8 Gy X-ray. Columns, mean; bars, ± SD; ^★^P<0.001, versus non-targeted shRNA transfected cells.

**Figure 4:**
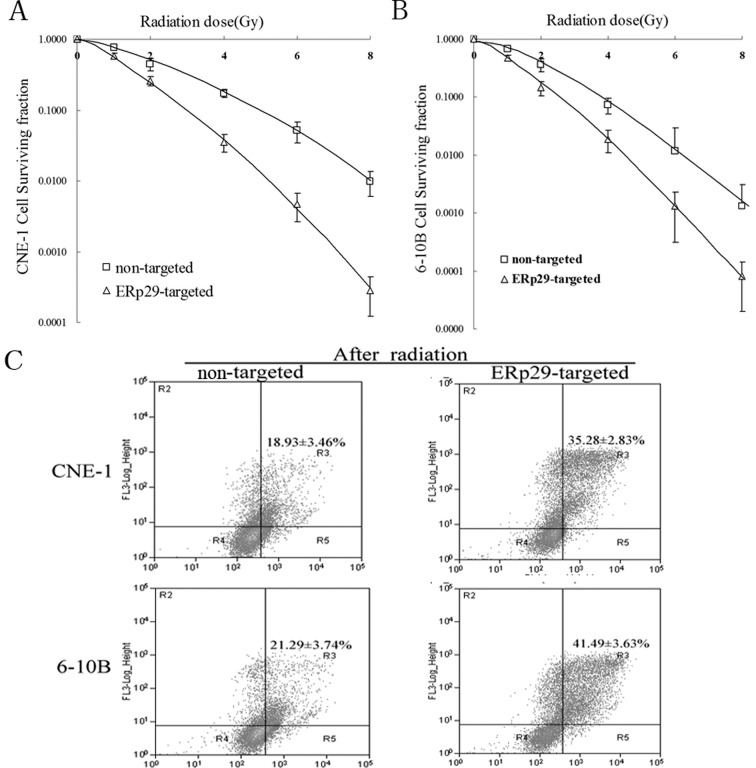
(A) The clonogenic survival curves of non-targeted or ERp29-targeted shRNA transfected CNE-1 cells. (B) The clonogenic survival curves of non-targeted or ERp29-targeted shRNA transfected 6-10B cells. Points, mean; bars, ± SD. (C) The apoptotic cells of non-targeted or ERp29-targeted shRNA transfected CNE-1 and 6-10B cells were determined by flow cytometry analysis at 72 h after irradiation with 8 Gy X-ray. Apoptotic cells (35.28±2.83%) were detected in ERp29 knockdown CNE-1 cells compared with apoptotic cells (18.93±3.46%) in controls, while apoptotic cells (41.49±3.63%) were detected in ERp29 knockdown 6-10B cells compared with apoptotic cells (21.29±3.74%) in controls.

NPC cells CNE-1 and 6-10B with higher ERp29 expression and stronger radioresistance were transfected with ERp29-targeted shRNA or non-targeted shRNA, and then cell viability assays and clonogenic survival assays were performed. Western blot analysis confirmed ERp29 knockdown in CNE-1 and 6-10B cells (Fig. 3A). Cell viability assays showed that CNE-1 and 6-10B cells transfected with ERp29-targeted shRNA resulted in a significant reduction of the cell viability at 48 h (P<0.001), as well as 72 and 96 h (P<0.001) after irradiation, comparing with the controls (Fig. 3B and C). In clonogenic survival assay, after ERp29 knockdown, SF2 and LD50 of CNE-1 cells ranged from 0.462 to 0.212 and 1.843 to 1.208, respectively, and SF2 and LD50 of 6-10B cells ranged from 0.324 to 0.144 and 1.142 to 0.875, respectively. In addition, the curve of ERp29-targeted shRNA CNE-1 cells was much steeper than the control (Fig. 4A). A similar situation was seen in 6-10B cells (Fig. 4B). Flow cytometric analysis showed transfected with ERp29-targeted shRNA CNE-1 and 6-10B cells resulted in a higher proportion of apoptotic cells comparing to transfected with non-targeted shRNA at 72 h (P<0.001) after irradiation (Fig. 4C). The result indicates that ERp29 up-regulation confers a significant protection against ionizing radiation and increases the resistance of NPC cells to X-ray radiation.

## Discussion

Proteome analysis of RR and RS tumor tissues allows the identification of aberrantly expressed proteins in cancer that might provide key information for identification of biomarkers predicting cancer radiosensitivity (22). To identify the proteins associated with the radioresistance of NPC, comparative proteomics was used to filtrate for differential proteins in the RR and RS NPC tissues. As a result, twelve differentially expressed proteins were identified. ERp29, a potential radioresistance-associated protein, was found significantly up-regulated in RR NPC tissues compared to RS NPC tissues.

To confirm the association of ERp29 with NPC radioresistance, immunohistochemistry was performed to detect the expression of ERp29 in the RR and RS NPC tissues as well as NPC cell lines with different radiosensitivity, and the correlation of its expression levels with NPC radioresistance was evaluated. The result showed that the expression level of ERp29 was positively related to the radioresistance of NPC tissues and cell lines. Furthermore, knockdown of ERp29 rendered CNE-1 and 6-10B cells more sensitive to radiation, which strongly indicated that ERp29 up-regulation plays an important role in the development of NPC radioresistance. These results demonstrate that ERp29 plays a protective role against radiation stress and is a factor inducing radioresistance in NPC.

ERp29 is a characterized resident of the cellular ER and it is expressed ubiquitously and abundantly in mammalian tissues (23). In most cases, ERp29 interacts with BiP/GRP78, an abundant ER-resident molecular chaperone, and this combination has been strengthened under ER stress (16,24). It was reported that ERp29 was up-regulated under conditions of homocysteine or dopamine invoked ER stress (25,26). In addition, when mouse intestinal epithelial cells were exposed to radiation, ERp29 was highly expressed and involved in ER stress (17) indicating that ERp29 is associated with resistance to oxidative and radiation stress and may play a potential protective role against stress.

Radiation therapy usually activates unfolded protein response (UPR) and results in the overaccumulation of malfolded, denatured or aggregated proteins in the cytoplasm (27,28). In response to UPR, XBP1 (a gene of ER stress sensor) was alternatively spliced by the activated endonuclease domain. Under these conditions, ER chaperones, ERp29, protein disulphide isomerase (PDI)-like proteins and GRP78, are up-regulated and accomplished by binding to denatured or aggregated cellular proteins to facilitate their refolding, thereby alleviating cell stress response (24,29). Besides, other molecules may also play a critical role in ERp29 mediated stress protection. A recent study found that ERp29 could potentiate resistance to doxorubicin by up-regulating Hsp27 in breast cancer cells through sequently down-regulating the α subunit of the eukaryotic initiation factor 2 (elF2α) (30), which can trigger apoptotic signals by activation of downstream molecule CHOP (31,32). While Hsp27 overexpression has been reported to inhibit apoptosis through direct inhibition of caspase activation (33–35).

In summary, we used proteomic approach identifying ERp29 up-regulated in the RR NPC tissues. We further showed that ERp29 contributes to NPC radioresistance and is a potential biomarker for predicting NPC response to radiotherapy. The findings reported here could have clinical value in distinguishing radiosensitive from radioresistant NPC and in identifying subgroups of NPC patients that could benefit from personalized therapeutic strategies.

## Figures and Tables

**Table I:** Clinicopathological parameters of the NPC tissue specimens.

Classification	Number
Gender	
Male	63
Female	25
Age	
≥50	27
<50	61
Histological type	
WHO type III	88
Primary tumor (T) stage	
T1	7
T2	38
T3	25
T4	18
Lymph node metastasis	
Negative	43
Positive	45
Distant metastasis (M)	
Negative	82
Positive	6
Clinical stage	
II	29
III	35
IV	24
Recurrence	
Negative	76
Positive	12

The NPC tissues were 88 cases of formalin-fixed and paraffin-embedded archival tissue specimens.

**Table II:** Differential expression proteins between RR and RS NPC identified by MALDI-TOF MS.

Spot no.	NCBInr Identification no.	Protein name	Score	Coverage (%)	Expression in RR/RS NPC
1	gi 20150229	Multidrug resistance-associated protein 14 (MRP-14)	150	91	↓
2	gi 38503339	Manganese superoxide dismutase (Mn-SOD)	73	52	↑
3	gi 5803013	Endoplasmic reticulum protein 29 (ERp29)	77	58	↑
6	gi 4503607	Human electron transfer flavoprotein	169	59	↓
7	gi 88191913	Thrombospondin-1 (TSP-1)	68	33	↓
8	gi 662841	Heat shock protein 27 (Hsp27)	68	47	↑
10	gi 31543380	Parkinson protein 7 (DJ-1)	88	52	↓
11	gi 66910342	Pyruvate kinase, muscle (PKM2)	72	39	↓
12	gi 4505773	Prohibitin	252	75	↓
14	gi 34783447	Glia maturation factor-γ (GMFG)	95	53	↓
15	gi 4758484	Glutathione S-transferase ω1 (GST ω1)	109	56	↑
18	gi 18999392	Cytochrome c oxidase	67	28	↓

**Table III:** The difference of ERp29 expression in the RR and RS of NPC tissues.

		Score			
			
	n	Low (0–2)	Moderate (3–4)	High (5–6)	P
Radioresistance	42	5 (11.9%)	13 (31.0%)	24 (57.1%)	<0.001
Radiosensitivity	46	31 (67.4%)	13 (28.3%)	2 (4.3%)	

P<0.05 was considered to be significant by χ^2^ test.
